# Metastatic squamous cell carcinoma neck with occult primary: A retrospective analysis

**DOI:** 10.4103/0971-5851.65334

**Published:** 2009

**Authors:** Pragya Shukla, Deepak Gupta, Shyam Singh Bisht, Mohan Chand Pant, Madan Lal Bhatt, Kirti Srivastava, Mahendra Pal Singh Negi

**Affiliations:** 1 *Department of Radiation Oncology, TATA Memorial Cancer Hospital, Mumbai, India*; 2 *Department of Radiation Oncology, Medicity Gurgaon, India*; 3 *Department of Radiotherapy, Chatrapati Shahuji Maharaj Medical University, Lucknow, Uttar Pradesh - 226 000, India*; 4 *Department of Biostats, Central Drug Research Institute, Lucknow, India*

**Keywords:** *Chemoradiotherapy*, *head and neck*, *metastatic cervical lymph node*, *radiotherapy*, *unknown primary*

## Abstract

**Introduction:**

Metastatic carcinoma in the lymph nodes of the neck from an unknown primary is relatively rare, accounting for about 3% of all head and neck cancers. Management of secondary neck of undetermined primary is controversial.

**Materials and Methods:**

The case records of all the patients treated in the Department of Radiotherapy, Chatrapati Shahuji Maharaj Medical University, from Oct 1999 to Sep 2004, were studied and the patients with secondary neck without a known primary tumor were analyzed in detail to elucidate the outcome of various treatment modalities in various stages of the disease. One hundred and forty patients were found to be eligible for this analysis. Initial treatment could be divided into two categories: concurrent chemoradiation (*n*=76) and radiotherapy alone (*n*=64).

**Results:**

The patients who had received radiotherapy alone (53.1%) had lesser complete response as compared to those who had received chemoradiotherapy (68.4%). The overall survival duration in patients of the radiotherapy treatment group ranged from 5 to 60 months, with an average (±SD) of 31.06 ± 21.01 months, while in the chemoradiotherapy treatment group it ranged from 6 to 60 months, with an average (±SD) of 39.42 ± 21.33 months. Both hematological and nonhematological toxicities, although higher in the chemoradiotherapy group, showed statistically insignificant differences.

**Conclusion:**

To the best of our knowledge, this is the only study evaluating the role of concurrent chemoradiation in cases of secondary neck with primary unknown. The improved response rates along with an increased survival (both disease free and overall) show the superiority of chemoradiotherapy in the management of such cases.

## INTRODUCTION

Metastasis to the lymph nodes of the neck with an occult primary is relatively rare, accounting for about 3% of all head and neck cancers. Metastasis of unknown primary on the whole carries a very poor prognosis, but in the head and neck region this is not the case.[1–4] The 5-year disease-specific survival rates range upwards to 74% in modern series, with overall survival rates being in the 40–66% range.[5–9]

Patients who have a metastatic neck lymph node will have their primary tumors discovered in more than 90% of the cases through a careful physical examination,[1] computed tomography (CT) and/or magnetic resonance imaging, endoscopy and biopsies[10] and, more recently, by elective tonsillectomies and newer imaging techniques such as positron emission tomography scans.[11] Metastasis in the upper and middle neck (levels I, II, III and V) are generally attributed to head and neck cancers, whereas level IV is often associated with primaries below the clavicle.

Extended field radiotherapy can be used to treat the potential primary site of origin in a case of head and neck. This aggressive approach, although associated with significant morbidity, has been advocated by many oncologic centers[12–17] and has also been challenged by others.[18]

In patients with early-stage neck disease (N1 or small mobile N2a disease), surgery alone can be used for treatment, but based on tenets established by Fletcher *et al*,[19] and expanded by Wang *et al*,[20] postop RT is recommended in patients when there is connective tissue invasion, multiple involved nodes or a suspicion of residual microscopic disease in the neck without clinically detectable tumor. Bataini *et al*.[13] had reported 48 patients treated with neck dissection followed by radiotherapy to head and neck mucosa and bilateral lymph nodes and 90 patients treated with radiotherapy alone. They had reported a 4% rate of developing carcinomas in head and neck mucosal sites Colletier *et al*.[5] had reported 136 patients treated with neck dissection followed by radiotherapy to head and neck mucosal sites and bilateral lymph nodes. In their study, 6% of the patients developed carcinomas in head and neck mucosal sites within radiotherapy portals and 4% of the patients developed carcinomas outside radiotherapy portals. The absolute survival achieved at 5 years was 60%. Reddy and Marks[17] had reported 16 patients treated with radiotherapy to ipsilateral lymph nodes and 36 patients treated with RT to head and neck mucosal sites and bilateral lymph nodes and had concluded that RT reduced the rate of developing head and neck cancer.

Management of secondary neck of undetermined primary is controversial as the best treatment for such cases remains unclear because of the heterogenous pathological condition. Various therapeutic regimens are present, but no clear-cut consensus has evolved. To assess the outcome of patients in a real-world situation, we retrospectively reviewed the database in our department to analyze the response rate, disease-free survival rate and overall survival rate of various treatment modalities along with an assessment of the associated toxicities.

## MATERIALS AND METHODS

The case records of all the patients treated in the Department of Radiotherapy of CSMMU (erstwhile KGMU) during a period of 5 years (Oct 1999 to Sep 2006) were studied and the patients with metastatic neck malignancies without a known primary tumor were analyzed in detail to elucidate the outcome of various treatment modalities like radiation and chemotherapy (singly or in combination) in various stages of the disease. Classification of a patient as having an unknown primary tumor was carried out if adequate investigations failed to detect a possible primary tumor site at the time when a final treatment decision was made. Twenty-six patients were excluded from the analysis on the basis of the following criteria: patients who were treated with palliative intent (*n* = 16), nonsquamous cell histology (*n* = 2) and took incomplete treatment (*n* = 8). All patients included in the study had cytologically proven squamous cell carcinoma, treated with curative intent, had no evidence of any distant metastasis and had not received any previous chemo or radiotherapy. Patients who had primarily been treated by surgical modality were not included in this analysis. Eventually, only 140 patients were found to be eligible for this analysis.

Of the 140 patients, 114 were men and 26 were women. Seventy-eight (55.71%) had a history of tobacco intake in some form or the other. Mean duration of symptoms was 3 months. Most common presentation was ipsilateral adenopathy. All patients had chest X-ray and panendoscopy performed as part of their work-up and 62 (44%) patients had a CT of the head and neck region. Other investigations performed were high-resolution ultrasonography of neck and biopsies. The work-up procedures were not significantly different between the different treatment groups. All available clinical and diagnostic information was used for clinical staging. For this report, all patients were nodal staged according to the AJCC, 1998 classification. Initial treatment of the patients could be divided into two categories: concurrent chemoradiation (*n*=76) and radiotherapy alone (*n* = 64). The patients had been regularly followed-up with endoscopy and/or imaging. For those patients for whom complete follow-up data was not available, contact was established with the help of phone calls and letters.

The median dose of radiotherapy in patients treated with radiotherapy alone was 67 Gy (range, 60–70 Gy) as opposed to a value of 66 Gy (range, 60–70 Gy) for the concurrent chemoradiotherapy arm.

RT was delivered with megavoltage equipment using parallel opposed lateral portals treating head and neck mucosal sites (the main potential primary sites of nasopharynx, oropharynx, larynx and hypopharynx) and upper cervical lymph nodes and *en face* anterior portals treating lower cervical and supraclavicular lymph nodes by once-daily fractionation. Chemoradiotherapy patients were given weekly IV Cisplatin in a dose of 35 mg/m^2^ along with radiation.

## RESULTS

### Statistical analysis

Continuous variables were compared by two-sample *t*-test while categorical variables were compared by the χ^2^ test. Difference between the two proportions was compared by proportion Z test using its correction for continuity. The Logrank test and Cox proportional hazard ratio were used to compare the two survival curves. A two-tailed (α = 2) probability (*P*) value less than 0.05 (*P*< 0.05) was considered to be statistically significant. Graph Pad Prism (version 5) and STATISTICA (version 7) were used for the analysis.

### Physical characteristics

Patient baseline demographic characteristics of both groups are summarized in Table 1. In both groups, the proportion of males was comparatively higher than that of females. Similarly, in both groups, stage N2b patients were the maximum and stage N3 were the minimum. On comparing all baseline characteristics of the patients in the two groups, they were found to be the same, i.e. they did not differ significantly (*P*>0.05).

**Table 1:** Baseline demographic characteristics of patients of both groups

Characteristics	Radiotherapy (*n* = 64)	Chemoradiotherapy (*n* = 76)	Statistic
Male (No.)	53 (82.8)	61 (80.3)	Z = 0.17^ns^
Female (No.)	11 (17.2)	15 (19.7)	Z = 0.17^ns^
Age (years) (mean ± SD)	52.24 ± 11.60	55.10 ± 61.40	T = 1.81^ns^
Node (No.)			
I	12 (18.8)	16 (21.1)	Z = 0.13^ns^
IIa	16 (25.0)	20 (26.3)	Z = 0.02^ns^
IIb	20 (31.3)	24 (31.6)	Z = 0.14^ns^
IIc	12 (18.8)	10 (13.2)	Z = 0.67^ns^
III	4 (6.3)	6 (7.9)	Z = 0.05^ns^

ns, *P*>0.05, Figures in parenthesis are in percentage

### Treatment response

The treatment responses, either complete or partial, in patients of both groups are summarized in Table 2. A complete response rate of 53.1% was seen in patients who had been treated with radiotherapy whereas the patients who had been treated with chemoradiotherapy showed a complete response of 68.4%. The complete response was 1.3-times higher in the chemoradiotherapy treatment group, but their proportions in the two groups did not differ significantly (53.1% *vs*. 68.4%, Z=1.68; *P*>0.05).

**Table 2:** Treatment response in patients of both groups

Response	Radiotherapy (*n* = 64) No. (%)	Chemoradiotherapy (*n* = 76) No. (%)
Partial response	30 (46.9)	24 (31.6)
Complete response	34 (53.1)	52 (68.4)

### Survival

#### Disease-free survival

The disease-free duration in patients of the radiotherapy treatment group ranged from 14 to 60 months, with an average (±SD) of 41.59 ± 18.43 months, while in the chemoradiotherapy treatment group it also ranged from 14 to 60 months, with an average (±SD) of 44.85 ± 17.29 months. The mean duration of disease free survival in patients of the chemoradiotherapy treatment group was 3.26 months higher than that in the radiotherapy treatment group, but the disease-free duration between both groups did not differ significantly (*P*>0.05), i.e. it was found to be the same (41.59 ± 18.43 *vs*. 44.85 ± 17.29; *t* = 0.83, *P*>0.05).

The disease-free proportion and its duration (months) in patients of both groups are summarized in Table 3. Of 64 patients in the radiotherapy treatment group, 34 (53.1%) patients were disease free and of 76, 52 (68.4%) were disease free in the chemoradiotherapy treatment group. In other words, the odds of disease free were 1.9-times higher in the radiotherapy treatment group as compared to the chemoradiotherapy group (OR = 1.912, 95% CI = 0.959–3.809; χ^2^ = 3.431, *P*>0.05).

**Table 3:** The number and disease-free duration (months) in patients of both groups

Disease-free survival	Radiotherapy (*n* = 64)	Chemoradiotherapy (*n* = 76)
No.	34 (53.1)	52 (68.4)
Mean ± SD	41.59 ± 18.43	44.85 ± 17.29
Median	42	54
Death	18 (52.9)	23 (44.2)
Live	16 (47.1)	29 (55.8)

Figures in parenthesis are in percentage

Similarly, of 34 disease-free patients in the radiotherapy treatment group, 16 (47.1%) patients were found alive at the final evaluation (6 years) while out of 52, 29 (55.8%) in the chemoradiotherapy treatment group were found to be alive. The proportion of survival was 1.2-times higher in the chemoradiotherapy treatment group, but the increase was found to be insignificant when compared with the survival proportion of the radiotherapy treatment group (55.8% *vs*. 47.1%; Z = 0.57, *P*>0.05). In other words, the odds of survival is 1.4-times higher in the radiotherapy treatment group as compared to chemoradiotherapy (OR = 1.418, 95% CI = 0.596–3.379; χ^2^ = 0.625, *P*>0.05).

**Figure 1:**
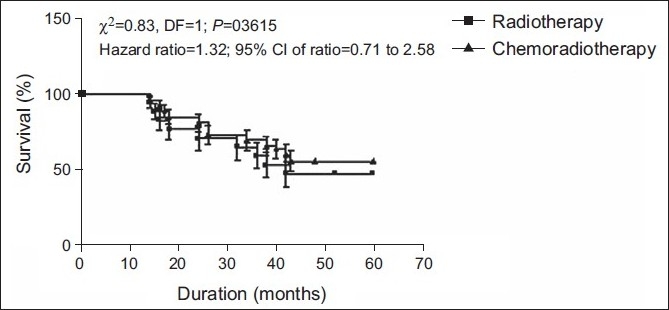
Disease-free survival (%) in patients

The disease-free survival (percent survival as a function of time) in patients of both groups was again analyzed by survival curve analysis and has been summarized graphically in Figure 1. The survival curve analysis also shows that the disease-free survival (%) between the two groups was the same (Log rank test: χ^2^ = 0.833; *P*>0.05). The hazard ratio indicates that the rate of death was 1.3-times higher in the radiotherapy treatment group as compared to the rate of death in the chemoradiotherapy treatment group (hazard ratio = 1.324; 95% CI of ratio = 0.708–2.575).

### Survival with recurrence

The recurrence duration in patients of the radiotherapy treatment group ranged from 14 to 52 months, with an average (±SD) of 28.70 ± 12.86 months, while in the chemoradiotherapy treatment group it ranged from 14 to 48 months, with an average (±SD) of 29.69 ± 11.48 months. The duration of recurrence was 1.0 month earlier in radiotherapy treatment group as compared to the chemoradiotherapy treatment group, but the duration of recurrence between the groups did not differ significantly (*P*>0.05), i.e. it was found to be the same (28.70 ± 12.86 *vs*. 29.69 ± 11.48, *t* = 0.28; *P*>0.05).

The recurrence proportion and its duration (months) in patients of both groups has been summarized in Table 4. Of 34 disease-free patients in the radiotherapy treatment group, the disease recurred in 20 (58.8%) patients while out of 52, 26 (50.0%) patients had disease recurrence in the chemoradiotherapy treatment group. The proportion of recurrence was 1.2-times higher in the radiotherapy treatment group as compared to the chemoradiotherapy treatment group, but the proportions between the groups was found to be the same (58.8% *vs*. 50.0%, Z = 0.58; *P*>0.05). In the radiotherapy group, six patients had recurrence at the primary site (three in oropharynx, two in hypopharynx, one in nasopharynx), 10 at the nodal site and four showed distant metastasis. In the chemoradiotherapy group, five (two in oropharynx, three in hypopharynx) patients had recurrence at the primary site, 12 at the nodal site and seven showed distant metastasis. Salvage surgery was used when feasible. For the remaining, adjuvant chemotherapy was given.

**Table 4:** The number and recurrence duration (months) in patients of both groups

Survival with recurrence	Radiotherapy (*n* = 34)	Chemoradiotherapy (*n* = 52)
No.	20 (58.8)	26 (50.0)
Mean ± SD	28.70 ± 12.86	29.69 ± 11.48
Median	28	26
Death	18 (90.0)	23 (88.5)
Live	2 (10.0)	3 (11.5)

Figures in parenthesis are in percentage

Similarly, of 20 patients showing recurrence in the radiotherapy treatment group, two (10.0%) patients were found to be alive at the final evaluation while of 26, three patients were found to be alive (11.5%) in the chemoradiotherapy treatment group. The proportion of recurrence survival was 1.2-times higher in the chemoradiotherapy treatment group, but found to be insignificant when compared with recurrence survival of the radiotherapy treatment group (11.5% *vs*. 10.0%; Z = 0.31, *P*>0.05). In other words, the odds of recurrence survival was 1.2-times higher in the radiotherapy treatment group as compared to chemoradiotherapy (OR = 1.174, 95% CI = 0.177–7.794; χ^2^ = 0.028, *P*>0.05).

**Figure 2:**
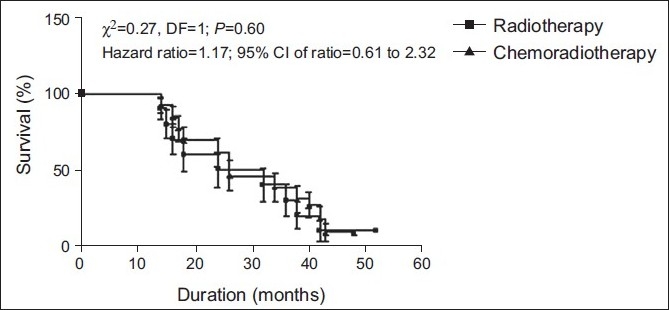
Recurrence survival (%) in patients of both groups

The recurrence survival (%) in patients of both groups has been summarized graphically in Figure 2. The recurrence survival between the two groups was found to be the same (Log rank test: χ^2^ = 0.274; *P*>0.05). However, the death rate in recurrence patients of the radiotherapy treatment group was 1.2-times higher than the death rate in the chemoradiotherapy treatment group (hazard ratio = 1.167; 95% CI of ratio = 0.614–2.324).

### Overall survival

The overall survival duration in patients of the two groups has been summarized in Table 5. The overall survival duration in patients of the radiotherapy treatment group ranged from 5 to 60 months, with an average (±SD) of 31.06 ± 21.01 months, while in the chemoradiotherapy treatment group, it ranged from 6 to 60 months, with an average (±SD) of 39.42 ± 21.33 months. The mean overall survival duration of the chemoradiotherapy treatment group was 8.36 months higher and significantly greater (*P*< 0.05) than the radiotherapy treatment group (39.42 ± 21.33 *vs*. 31.06 ± 21.01, *t* = 2.33; *P*< 0.05).

**Table 5:** The overall survival duration (months) in patients of both groups

Overall survival	Radiotherapy (*n* = 64)	Chemoradiotherapy (*n* = 76)
Mean ± SD	31.06 ± 21.01	39.42 ± 21.33
Median	21.5	43
Death	48 (75.0)	47 (61.8)
Live	16 (25.0)	29 (38.2)
Live	2 (10.0)	3 (11.5)

Figures in parenthesis are in percentage

Of 64 patients in the radiotherapy treatment group, 16 (25.0%) patients were found to be alive at the final evaluation while out of 76, 29 (38.2%) patients were alive in the chemoradiotherapy treatment group. The overall survival was 1.5-times higher in the chemoradiotherapy treatment group, but the increase was found to be insignificant when compared with the radiotherapy treatment group (25.0% *vs*. 38.2%; Z = 1.48, *P*>0.05). In other words, the odds of overall survival was 1.9-times higher in the radiotherapy treatment group as compared to the chemoradiotherapy group (OR = 1.851, 95% CI = 0.891–3.846; χ^2^ =2.758, *P*>0.05).

**Figure 3:**
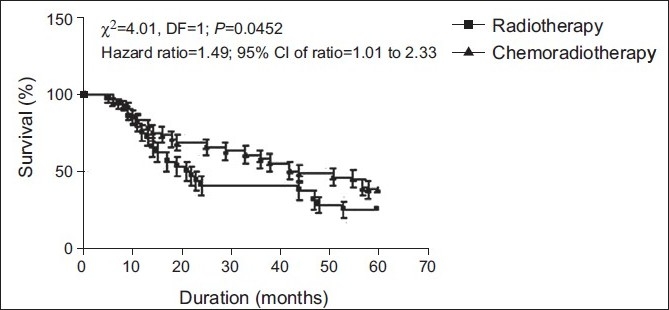
Overall survival (%) in patients of both groups

The overall survival (%) in patients of both groups has been summarized graphically in Figure 3. The overall survival in patients of the chemoradiotherapy treatment group was found to be significantly (*P*< 0.05) higher than the radiotherapy treatment group (Log rank test: χ^2^ =4.013; *P*< 0.05). In other words, the death rate was 1.5-times higher in the radiotherapy treatment group than the death rate in the chemoradiotherapy treatment group (hazard ratio = 1.493; 95% CI of ratio = 1.009–2.325).

### Toxicity

Grade III and IV hematological and nonhematological toxicities are summarized in Table 6. The grade III and IV toxicities were found to be higher in the chemoradiotherapy group as compared to the radiotherapy group. However, this difference is not found to be statistically significant (*P*>0.05).

**Table 6:** Grade III/IV acute (nonhematological and hematological) toxicity in both groups

Toxicity	Chemoradiotherapy arm (*n* = 76)	Radiotherapy arm (*n* = 64)	*P*
Nonhematological			
Mucositis	40 (53)	26 (41)	0.4525
Skin	13 (17)	7 (11)	0.4725
Nausea	18 (24)	10 (16)	0.406
Vomiting	5 (7)	2 (3)	0.4594
Diarrhea	4 (5)	0 (0)	0.1291
Hematological			
Anemia	10 (13)	5 (8)	0.422
Thrombocytopenia	3 (4)	0 (0)	0.2530
Neutropenia	5 (7)	0 (0)	0.07

## DISCUSSION

Lymph node metastasis to neck from the occult primary in the head and neck region is rare. Large reported series indicated that the frequency is around 3% of the total head and neck cancer cases.[1–4] Owing to the rarity of the disease, all reports on treatment results of patients with squamous cell carcinoma of unknown primary tumor site presenting with cervical neck nodes are retrospective.

The retrospective nature of our report is its limitation, but has an edge because of the larger number of patients and higher incidence of advanced nodal disease to those of other literature series; the most frequent lymph node 73 *vs*. 57% of patients had N2 stage and 16 *vs*. 10% of the cases had bilateral neck node involvement.[21–23] Also, to the best of our knowledge, this is the first analysis evaluating the role of concurrent chemoradiation in such patients.

The mean age in our study was 54 years. The mean age at diagnosis has varied in series from 55 to 65 years, and the younger median age in some series may partially be explained by the inclusion of undifferentiated tumors. Likewise, the male preponderance in our study is very well in consistence with that reported in previous studies on head and neck carcinoma.[24]

Comparing the diagnostic workup performed in our patients with that of the Danish National Study[25] in which all patients underwent panendoscopy with random biopsies of the upper aerodigestive mucosa,[11] this procedure was not systematically performed in all our cases.

The prognosis of unknown primary with cervical metastasis is similar to that of patients with known primary head and neck carcinoma and an identical N category, with up to 50% of these patients being long-term survivors.[26,27] This reasonable prognosis is crucial in determining justification for an aggressive management. However, in the absence of randomized trials, the most favorable treatment approach has not, until now, been defined. The proposed treatment options for neck metastases include neck dissection alone, radiotherapy alone or neck dissection with postoperative radiotherapy. A review of the literature suggests that the most promising results have been achieved with neck dissection followed by comprehensive irradiation whenever feasible.[28,29]

Evaluation of the survival obtained by the use of concurrent chemoradiotherapy and radiation in patients of cervical metastasis from an unknown primary was the aim of our study. The 5-year overall survival of our series was 31.6%, which is in accordance with that reported in the literature, analyzing mainly patients with lymph node metastases with squamous cell carcinoma, varying from 22% to 67%.[5,17,30–33] Studies on carcinoma head and neck in the past have established the role of concurrent chemoradiotherapy because of the achieved survival advantage.[34–38] However, patients of secondary neck with primary unknown were excluded from most of these studies. In a phase II study, Jeremic *et al*. had reported that 12 of 21 patients with metastatic squamous cell carcinoma of an unknown primary tumor localized to the neck and who were treated with bilateral neck and mucosal irradiation and concurrent weekly Cisplatin were found to be without evidence of disease.[39]

The neck nodes (22.5%), followed by distant metastases (16.1%) and primary tumor (15.8%), were the most frequent sites of relapse in our study. This is in accordance with previous studies that have shown that the most frequent site of recurrence was neck nodes, followed by distant metastases.[5,23,25,32,40] Colletier *et al*.[5] had shown a neck node recurrence rate of 9% with a distant metastasis rate of 18% and onset of primary tumor of 14%. However, Reddy and Marks[17] found that the rate of relapse at the primary tumor site was higher than that of nodal recurrence.

The most frequent site of primary tumor occurrence was the oropharynx, followed by the nasopharynx, hypopharynx and the larynx. The most frequent sites that have been reported in the literature were oral cavity, oropharynx, nasopharynx and larynx.[21,25] The incidence of distant metastases in our series (16.1%) was similar to that of other studies, in which incidence varied from 11% to 33%.[41,42]

The nonhematological toxicities of mucositis, skin, nausea, vomiting and diarrhea were 41%, 11%, 16%, 3% and 0%, respectively, in the radiotherapy arm and 53%, 17%, 24%, 7% and 5% in the chemoradiotherapy arm. The difference was not statistically significant. Similarly, hematological toxicities like anemia, thrombocytopenia and neutropenia in the chemoradiotherapy arm were 8%, 0%, 0% and 13%, 4%, 7% in the radiotherapy arm. The difference again was not significant. Late toxicities like dysphagia, xerostomia and neck fibrosis in the chemoradiotherapy arm were 6.6%, 9.2%, 7.9% and 6.2%, 7.8%, 7.8% in the radiotherapy arm, the difference being statistically insignificant. The toxicity values of both acute and late reactions is similar to those that have been reported previously.[24]

Our study too has shown that patients who had been treated with concurrent chemoradiotherapy fared better than those who had received radiotherapy alone. The toxicity, although higher in the chemoradiotherapy arm than in the radiotherapy-alone arm, was not found to be significantly different.

## CONCLUSION

The improved response rates along with an increased survival (both disease free and overall) at manageable toxicity clearly show the superiority of chemoradiotherapy in the management of secondary neck with primary unknown.
